# Supplementary figures and images for: Analogous computations in working memory input, output and motor gating: Electrophysiological and computational modeling evidence
Source: PLoS Comput Biol. 2021 Jun 7;17(6):e1008971. doi: 10.1371/journal.pcbi.1008971 (PMC8211210; doi:10.1371/journal.pcbi.1008971)

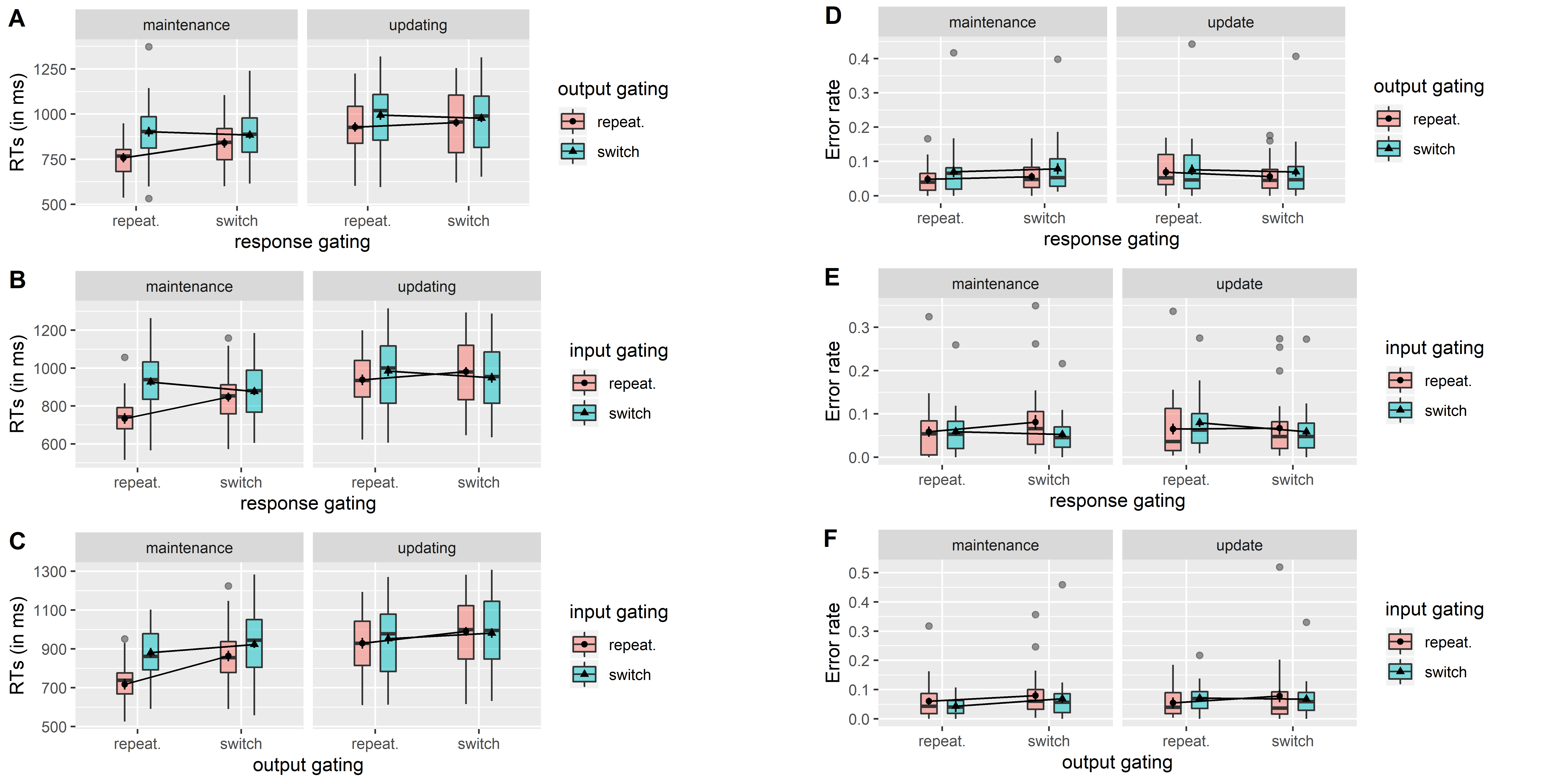

Supplement: S1 Fig — Mean RT (A-C) and Error rate (D-F) demonstrate differences in performance across all levels of gate switching and interactions between gating levels (output and response, input and response and, input and output) in maintenance trials (left panels) and updating trials (right panels). (TIF) [file pcbi.1008971.s001.tif]

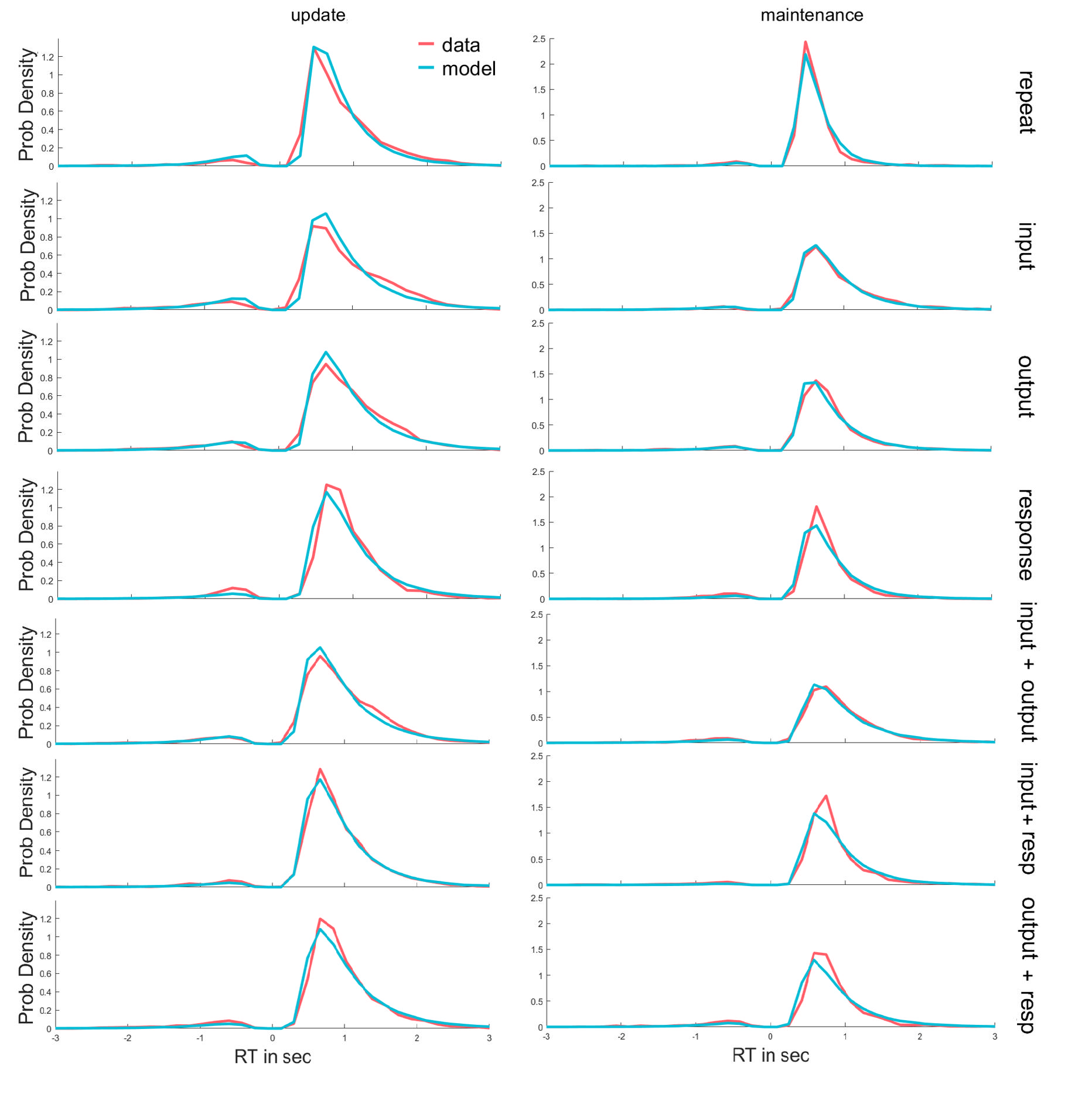

Supplement: S2 Fig — Behavioral RT distributions across the group are shown for switching at each level (red line) together with posterior predictive simulation from the HDDM (light blue). Distributions of correct (the right positive tail) and incorrect (left negative tail) trials in updating trials (left) and maintenance trials (right) show good correspondence between data and model. (TIF) [file pcbi.1008971.s002.tif]

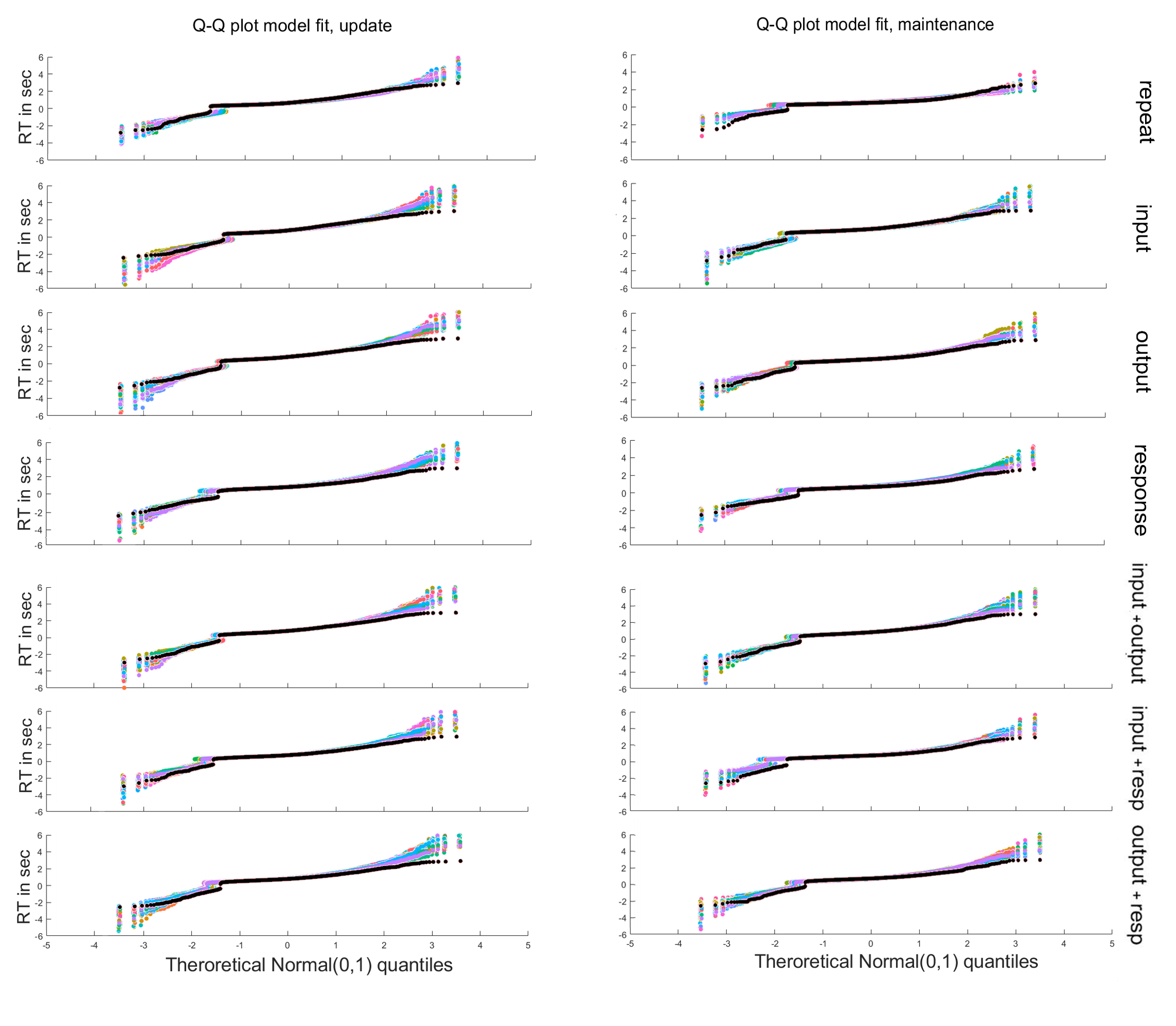

Supplement: S3 Fig — Model fit to behavior can be more precisely viewed using quantile-quantile plots, showing quantiles in updating (left) and maintenance (right) trials, of the empirical behavioral RT distributions (black) against the 50 simulation of RT distribution (colored lines, capturing model uncertainty) from the posterior predictive of the HDDM model, for correct response (positive RT) and incorrect responses (negative RT). Quantiles were computed at the group level. (TIF) [file pcbi.1008971.s003.tif]

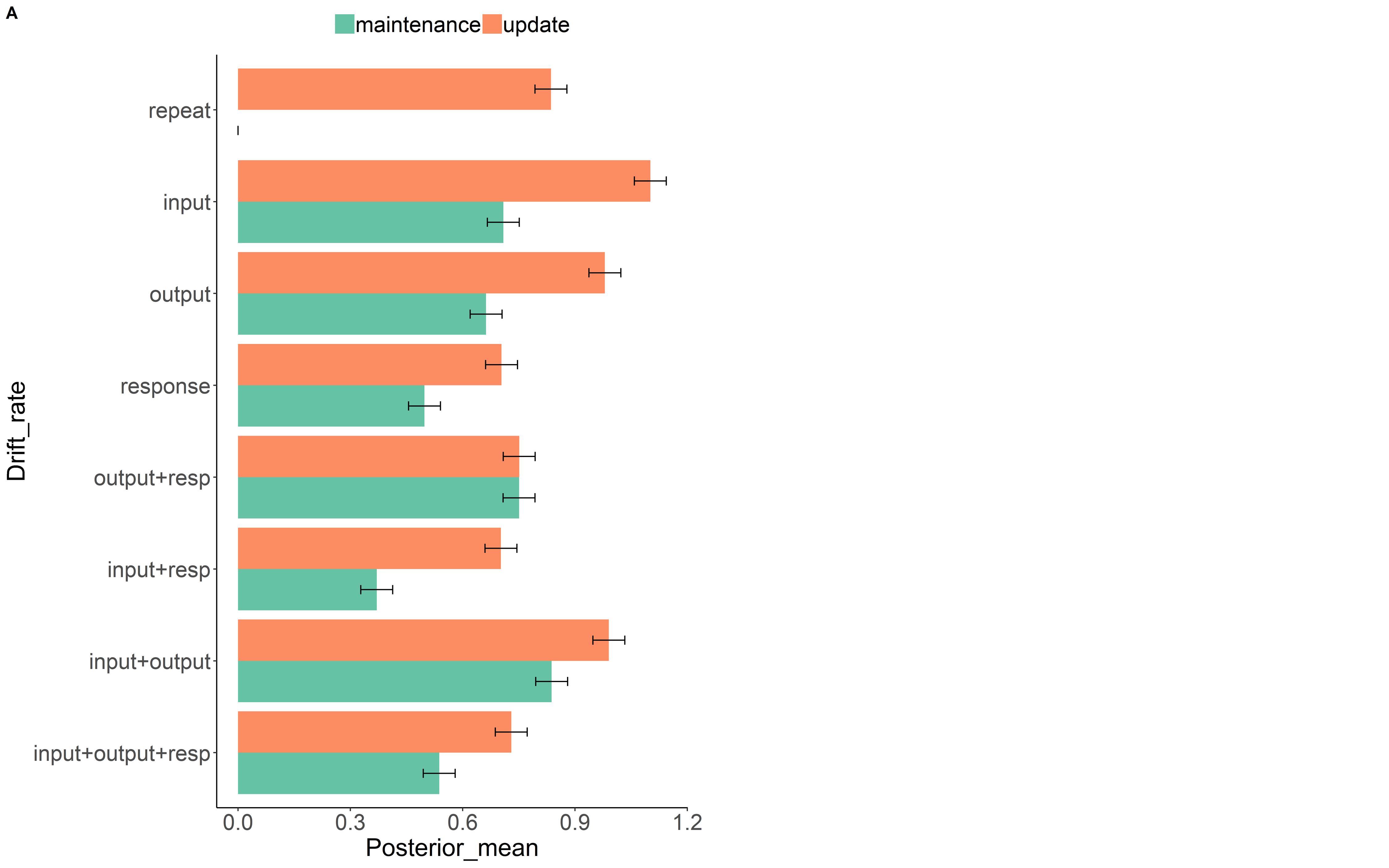

Supplement: S4 Fig — Drift rate in updating (orange bars) and maintenance trials (green bars) exhibit slowing (lower drift) in conditions that required more cognitive processing and exhibit facilitative interactions between gate switches. A possible interpretation for the facilitation finding is that WM updating decisions increase the mutually facilitative effect of switching across the gating system. The relative drift rates are presented as positive for plotting convenience. (TIF) [file pcbi.1008971.s004.tif]
