# Supplementary material for: Analogous computations in working memory input, output and motor gating: Electrophysiological and computational modeling evidence
Source: PLoS Comput Biol. 2021 Jun 7;17(6):e1008971. doi: 10.1371/journal.pcbi.1008971 (PMC8211210; doi:10.1371/journal.pcbi.1008971)
Supplement: S1 Text — (DOCX) [file pcbi.1008971.s005.docx]

S1 Text

Supporting Information. The extended behavior results

A five repeated-measures ANOVA on mean RT with updating policy (update, maintenance) × input gating (switch, repeat) × output gating (switch, repeat) × response gating (switch, repeat) × updating frequency (frequent updating, rare updating) revealed significant main effects for WM policy (*F*_1,29_= 71.03, *p*<.001, *η_p_^2^*=.71) with faster RT in maintenance trials than in updating trials (see S1 Fig) reflecting the additional time required to update WM with the presented stimulus. Significant main effects for input (*F*_1,29_= 68.75, *p*<.001, *η_p_^2^*=.70) and output gating (*F*_1,29_= 49.82, *p*<.001, *η_p_^2^*=.63) were also observed, with a marginal effect for response gating (*F*_1,29_= 4.18, *p*=.05, *η_p_^2^*=.13). Finally, the updating frequency had no significant main effect on behavior (*F_1,29_=* *0.84, p=.37, η_p_^2^=.03)* but it had a significant interaction with updating policy *(F_1,29_= 63.78, p<.001, η_p_^2^=.69)* suggesting that the slowing in updating compare to maintenance trials (updating cost) is reduced when updating is frequent and maintenance trials are rare.

The repeated-measures ANOVA also showed significant under-additive interactions between gating levels in the hierarchy, namely between input × output gating (*F_1,29_=* *38.13, p<.001, η_p_^2^=.57),* between output × response gating (*F_1,29_=* *21.43, p<.001, η_p_^2^=.42)* and between input × response gating (*F_1,29_=* *73.50, p<.0001, η_p_^2^=.72).* The three-way interaction between the three gates was also significant *(F_1,29_=* *13.53, p<.001, η_p_^2^=.32)* and so was the four-way interaction between updating policy × input × output × response switching (*F_1,29_= 9.98, p=.004, η_p_^2^=.26*) suggesting that the under-additive interactions between two gate switches is stronger in maintenance than in updating trials and is limited to the case where the third level is not switching.

In error rate, none of the main effects was significant (*F_1,29_<3.11)* but the two-way interactions between WM state × response (*F_1,29_= 6.68, p=.02, η_p_^2^=.19)* and input × response *(F_1,29_= 13.12, p=.001, η_p_^2^=.31)* were significant and so was the four-way interaction between updating policy × input × output × response switching *(F_1,29_= 14.14, p<.001, η_p_^2^=.33)*.

The significant four-way interactions in mean RT and error rate were further analyzed by running two separate three-way repeated-measures ANOVAs, once for updating trials and once for maintenance trials on mean RT and error rate with input gating (switch, repeat) × output gating (switch, repeat) × response gating (switch, repeat).

In updating trials, only output gating had a main effect (*F*_1,29_= 39.92, *p*<.001, *η_p_^2^*=.53) while input and response switching did not show a main effect (*F*_1,29_<1.1). However, the under-additive interactions between gating levels in the hierarchy, were all significant. Namely between input × output gating (*F_1,29_=* *12.09, p=.002, η_p_^2^=.29),* between input × response gating (*F_1,29_=* *13.33, p=.001, η_p_^2^=.31)* and between output × response gating (*F_1,29_=* *14.52, p<.001, η_p_^2^=.33).* The three-way interaction between the three gates was not significant *(F_1,29_=* *2.74, p=.21, η_p_^2^=.09).*

In maintenance trials, significant main effects were observed for input (*F*_1,29_= 187.58, *p*<.001, *η_p_^2^*=.87), output gating (*F*_1,29_= 54.83, *p*<.001, *η_p_^2^*=.65) and for response gating (*F*_1,29_= 12.96, *p*=.001, *η_p_^2^*=.31). Significant under-additive interactions between gating levels in the hierarchy were also observed between input × output gating (*F_1,29_=* *73.66, p<.001, η_p_^2^=.72),* between input × response gating (*F_1,29_=* *124.29, p<.001, η_p_^2^=.81),* and between output × response gating (*F_1,29_=* *24.60, p<.001, η_p_^2^=.46)*. The three-way interaction between the three gates was also significant *(F_1,29_=* *25.62, p<.001, η_p_^2^=.47)* suggesting that the under-additive interactions between two gate switches is limited to the case where the third level was not switching.

In updating trials, significant effects in error rate were observed only for the interactions between input × response gating (*F_1,29_=* *9.85, p=.004, η_p_^2^=.25),* and for the three-way interaction between the three gates *(F_1,29_=* *6.69, p=.02, η_p_^2^=.19)* suggesting that the under-additive interaction between input × response gating is limited to the case where output gate was not switching. None of the main effects nor the other two-way interactions were significant (*F_1,29_<2.7, p>.11).*

In maintenance trials, significant main effects were observed in error rate for input (*F*_1,29_= 4.28, *p*=.05, *η_p_^2^*=.13), output gating (*F*_1,29_= 6.39, *p*=.02, *η_p_^2^*=.18) and for response gating (*F*_1,29_= 6.27, *p*=.02, *η_p_^2^*=.18). The only significant interaction was between input × response gating (*F_1,29_=* *15.00, p<.001, η_p_^2^=.34),* where response switching was more accurate than response repetition when input was also switching.
