# Supplementary material for: Analogous computations in working memory input, output and motor gating: Electrophysiological and computational modeling evidence
Source: PLoS Comput Biol. 2021 Jun 7;17(6):e1008971. doi: 10.1371/journal.pcbi.1008971 (PMC8211210; doi:10.1371/journal.pcbi.1008971)
Supplement: Attachment — Submitted filename: Reviewers comments final.docx [file pcbi.1008971.s006.docx]

Responses letter

We would like to thank the reviewers for their thoughtful comments, evaluations and suggestions. In the attached response letter, we address each of the comments and highlight all the changes that were made to the ms. We put a special emphasis on increasing the interpretability of our results and figures in accordance with the suggestions made by the reviewers. We made significant changes to our manuscript and specifically on explaining the motivation behind our key predictions and for using the mathematical model, the hierarchical drift-diffusion model.

**Reviewer #1:** “Analogous computations in working memory input, output and motor gating: Electrophysiological and computational modeling evidence,” Rac-Lubashevsky and Frank. This is an interesting and carefully carried out EEG study of the “reference-back-2” working memory task that uses quantitative fits to the PBWM model to derive EEG correlates of gating operations specified by PBWM. This work makes a potentially very impactful contribution to the study of the neural bases of cognitive control because, as the authors state in the Introduction, “to date the gating WM literature and the WM prioritization literatures have not been linked.” Indeed, this statement could have been lifted from a grant proposal submitted by this reviewer just two months ago. My overall assessment is that I’m very enthusiastic about this work. However, there are several points where the exposition could be clearer, and some where the authors might consider making contact with other literature.

Overall, the paper would be easier to read if the Introduction were more coherent with the rest of the manuscript. For example, there are several constructs and/or distinctive empirical patterns that pop up in the Results section that relate to ideas introduced in the Introduction, but for which different words are used. For example, in the Intro we read that “switches give rise to a transient period of conflict;” but only in the Results are we told why: “In PBWM, gating signals are used to displace prefrontal activity states with new information.”

We thank the reviewer for this excellent point and we agree. We made more detailed explanations in the introduction to better motivate the results and to link to the additional literature as suggested in the next comments.

e.g., on page 7 (in the clean ms), the first key prediction was modified:

“Here, we aim to test four key predictions from PBWM. First, we assessed whether input, output, and response gating share analogous computations that evolve dynamically in time. In particular, in PBWM, gating signals are used to displace prefrontal activity states with new information. Thus, when gating demands (at any level) switch from one trial to the next, a transient period of conflict ensues in which the previous and currently relevant information are competing. As such, PBWM predicts gate switching will be associated with an independent response time cost that arises from PFC conflict analogous to that experienced when needing to switch motor responses in PMC [33] (see also Fig. 4 below). Indeed, all levels of conflict are thought to recruit the same basal ganglia mechanism that slows down responses and increases decision threshold [12, 33]. This prediction is also consistent with findings in which switching between objects in WM during cognitive operations gives rise to a performance cost [34-35]. In that literature, the slowing in switch trials is attributed to “attentional control”: the need to move the focus of attention to the other object in WM. Under this view, attention is allocated to only one process at a time thus creating a bottleneck that controls which WM representation to select (i.e., prioritize) and use to guide subsequent cognitive operations or action selection [36-37].”

This same section of the results introduces the importance of under-additive interactions, and these are illustrated in Figure 3. Only further along in this section is it explicitly stated that hierarchical gating processes that “evolve partly in parallel lead to under-additive interactions.” This is an important pattern in the data that returns several times, and its significance really should have been introduced in the portion of the Introduction that introduces the architecture of the PBWM. Additionally, the at-face-value contradiction of a hierarchically organized set of operations and “partly” parallel engagement needs to be explained more explicitly. E.g., even later we will encounter HDDM modeling of gating operations; is it perhaps possible that there is leakage from the decision that’s underway at, say, the input gating level that gets the output gating operation started before input gating is finished? Whether or not this is true, there are presumably quantifiable dynamics in PBWM that would provide a more rigorous level of understanding than just the verbal statement that some of these processes can be “partly” parallel.

We added a new key prediction to the ms to make the prediction about the expected temporal order explicit (see also page 9, in the clean ms):

“Third, we tested the prediction that input, output and response gating are processed in parallel. Converging evidence from neural network modeling of hierarchical rule learning [11,12, 51], and experimental work with cognitive-control tasks (e.g., [64-66]) suggest that WM and action selection are managed by independent mechanisms, operating mostly in parallel on different levels of information. In biologically inspired models of PBWM [11,12,51], parallel processing can occur when PFC projections to lower level striatum are strong, resulting in faster accumulation of evidence across levels of the hierarchy to favor a single response. The evidence for parallel processing across levels of information is also well documented in task switching literature when task sets and responses both switch [64-65] but also when context and items in WM both switch [66]. Parallel processing in these cases is signified by under-additive interactions reflecting that the cost of switching two levels of decision was less than the sum of their individual durations.

However, the PBWM modeling simulations and EEG work suggest that processing is not perfectly parallel: indeed, conflict about the task-set in PFC has to be resolved before motor action selection can be informed by the task-set. In the model, the degree of this influence is controlled by projections to the STN leading to an increase in decision threshold that prevents interference in stimulus-response mappings across task-sets [12]. Relatedly, in EEG data, switches in higher level task rules gave rise to anterior EEG signatures which precede those of more posterior motor EEG signatures [33]. Therefore, while there is evidence for simultaneous accumulation of evidence for decision across levels, there is also evidence for serial termination where response decision is delayed by conflict at the higher cognitive level. We aimed to assess whether neural markers of input, output and response gating are separately detectable, and whether their temporal order will also exhibit this partly parallel dynamic.”

(Additionally, whatever the answer is, it’d be helpful if an illustration of these dynamics could also be incorporated into Figure 1; perhaps a timeline of when different elements of the model are engaged?)

**“Figure 1. Schematic of the PBWM architecture for canonical gating operations**.

(a) The gating system is composed of three core circuits that are hierarchically arranged from left to right. For illustration the operations needed to solve the task used in this paper are shown. At the beginning of a trial, the memory layer actively maintains the information from the previous trial (e.g., “O” and “%”). Sensory information (e.g., the “X” in the visual input) is transiently represented in a prefrontal cortex layer (labeled “PFC-in”). The input gate controls whether the new visual information (e.g., “X”) is updated to be maintained in a PFC memory layer, and if so, to which address or anatomical “stripe” within that layer (selective gating). The colored frame in the visual input represents a task cue signifying the updating policy that should be taken by the input gate. In this example, the red frame indicates that memory should be updated, so the input gate selectively updates the right PFC stripe (corresponding with the letter category, replacing the “O” in memory with “X”). The output gate controls which memory representations is prioritized in PFC-out (e.g., a deep layer of the PFC) to influence subsequent processing (e.g., here the relevant representation in memory is “O” and not “%”, given that the input to be compared is in the letter category). The response gate controls which motor response to select (e.g., here “same” (S) or “different” (D)) in posterior prefrontal layer (labeled here PMC), by comparing the output-gated memory representation (“O”) with the current visual input (“X”). Gate switching at the output and response gates are reflected by the change in the active stripe between the previous trial (t-1) and in the current trial. **(b)** An example of a maintenance trial. The blue task cue indicates that the appropriate updating policy is maintenance, and thus sensory information (e.g., “X”) is prevented from being updated in PFC memory (the gating signal from the BG is therefore a No-Go, indicated by the red BG). Gate switching in the input gate corresponds with moving between updating state (as shown in a) and maintenance state (as shown in b). Yellow squares indicate active stripe selected by the corresponding gate. The orange square represents the newly updated stripe in memory. The predicted temporal order of the gating loops is depicted at the bottom right of the figure with input and output gating starting roughly at the same time continuing in parallel (indicated in orange) with output gating terminating later (serial termination indicated in grey). The response gate is expected to initiate with some delay due to conflict at upper levels, and to terminate even later than the output gate.

It strikes me as backward to say that “WM prioritization is thought to support ‘controlled attention’ by enhancing top-down influence…” Isn’t WM prioritization itself an example of controlled attention?

We agree and we modified the sentence (its location was also moved to the description of the first prediction): “In that literature, the slowing in switch trials is attributed to “attentional control”: the need to move the focus of attention to the other object in WM. Under this view, attention is allocated to only one process at a time thus creating a bottleneck that controls which WM representation to select (i.e., prioritize) and use to guide subsequent cognitive operations or action selection [36, 45].”

Is “BG gating” (p. 9) different from “gating”?

No, we corrected this sentence to highlight the fMRI results while avoiding such confusion (now of page 5 in clean ms):

“Behavioral, EEG and fMRI data support the notion that the reference-back task taxes cortico-striatal input gating.”

Contact with other literature. I’m hesitant to write this, because the authors already do a good job of citing a lot of the relevant literature, but there are some additional areas where contact can be made with work that one doesn’t necessarily classify as “PLoS Computational Biology”. The first is that the reference-back-2 is reminiscent of the “Garavan task,” a running span task that Hugh Garavan published back in the 90s(?) that, for a while at least, captured a lot of interest in the cognitive psychology of cognitive control. A second oldie is Marcia Johnson’s MEM, particularly the ‘refresh’ operation in relation to the increased decodability described here. Indeed, one of the things that so appealing about models like PBWM is that they posit explicit quantifiable mechanisms that can either ‘confirm’ and elaborate on older verbal models (as may be the case here with ‘refresh’), or, in other cases, provide better accounts. More recently than MEM, studies of retrocuing effects in working memory, some of them already cited, are germane.

Thanks for this. Please see previous responses for how we have now incorporated this literature in the introduction. In response to relevance of the ‘refresh’ operation to decodability we added the following to the description of our second prediction in the introduction (Page 8 in clean ms):

“Second, we tested the core PBWM prediction that output gating is used to enhance the representation associated with the currently relevant category (bolded representation in Fig 2). Previous work has shown that focused attention to one WM item prioritizes it. Directing attention to a specific WM content during the retention interval using an external cue (retro-cue; see review by [38]) or by instructing participants to refresh (“think of”) [39-40] puts such content in a privileged state where it is more resistant to perceptual interference, has better memory strength (reduced errors at retrieval) and heightened accessibility for later use [36, 38, 41, 42]”.

One question I’m thinking about came up in a finding of ‘enhanced decodability’ that Sprague and Serences interpreted as evidence for reactivation of ‘activity-silent’ components of a stimulus representation, but that others argued might just be akin to the sharpening one can see in bump attractor models when more energy is added. Do the present findings shed light on this, or is there perhaps a ‘future direction’ with this approach that could do so?

We agree that there are multiple potential mechanistic interpretations for what gives rise to the enhanced decodability, and we do not think our data strongly favors one or the other. However, we note that the PBWM model motivating our work does not rely on activity-silent components, in that there is ongoing active maintenance of both stimulus representations, but only one of them is prioritized due to output gating of deeper PFC layers which then amplify corresponding activity, making it more decodable (we allude to this in the Discussion). While we have simulated this mechanism with PBWM, it remains a proof of principle only and we thus do not wish to make a strong case based on our data here.

Finally, thinking about Figure 4, is there a way to relate any of the “gating components” extracted by the model to more classical ERP components, or perhaps to something like frontal midline theta?

ERP plots were added to the Figure with the topo plots:

The following discussion of the ERP components was added to the ms (page13 in clean ms):

“A closer look at these univariate patterns showed distinct ERP components for each level of gating at sites where the univariate patterns had maximal activity (Fig 4d). Specifically, the switch in the input gate to WM in updating trials showed increase in amplitude in the parietal P3b [63]. The same parietal site also showed a positive effect for response switching but at a later time window in the positive slow wave (PSW) component [64]. Finally, output switching was associated with a frontally negative enhancement in the N2 time range [65]. We speculate about the role of these ERP effects in the Discussion.”

In the general discussion (page 18 in clean ms) we explained the possible interpretations of these ERP components to gating:

“An ERP analysis of selected electrodes where the observed mass-univariate response was maximal, showed that each gating decision was marked with a different ERP component, suggesting again that each gating decision engages a separate mechanism. Specifically, input switching to an updating policy was marked by an increase in the P3b component, possibly reflecting the change in the relevance of the perceptual stimulus for the updating of WM (triggered by the switch in the task cue) [63-64]. This is consistent with previous work with the reference-back that has linked the P3b component to the categorization of the stimulus as goal-relevant [28] or as meaningful for behavioral adaptation in a context-specific manner [73]. The N2-like enhancement during output switching potentially reflects the increased need for response monitoring [65] induced by the conflict in the output gate. Such interpretation would be consistent with the predicted dependency between hierarchical gating loops such that conflict at the higher level output gate may also temporarily increase response conflict [12]. Finally, response switching was marked by an increase in the posterior PSW amplitude, possibly reflecting difficulty in selection or preparation of an action that is different from that already planned or still in memory from the previous trial [64, 74].”

Other comments
- There’s reference to RL in the intro, but no apparent explicit role for RL in the Results. But presumably there are prediction errors associated with many of the operations carried out in performing this task. Even if they’re not explicitly modeled here, some consideration of how they would be incorporated into a more complete simulation would be useful? This is also another chance to make contact with other literature, in that the model that underlies the Olivers and Roelfsema (2020) commentary (which is cited) very explicitly incorporates principles of RL on a trial-by-trial basis.

The PBWM model articulates how RL supports appropriate gating policies at all levels when such policies are not instructed. But in the current task all rules for correct responding, including when to input and output gate, are instructed. We do think RL processes nevertheless get engaged as a subject improves in the task such that their gating policies match the instructions (see e.g., Frank & O’Reilly 2006 and Moustafa et al 2008 for examples of how such gating policies can be learned and revealed after switches in instructions, and subject to dopaminergic modulation). However, it is difficult to access the prediction errors that would result from such operations without manipulating reward feedback and instructions in the task.

- The comparisons of 2-back vs. reference-back-2 tasks feel forced. For example, on one hand, reference-back-2 could also be thought to require “encoding, inhibition, binding, matching, maintenance, updating and removal,” and on the other hand, these operations derive from intuition, but people have successfully trained 3-layer RNNs to perform n-back tasks, and these models don’t explicitly implement most of these operations.

The reason we made the comparison to the n-back task is because it is the most commonly used WM updating task, and its continuous nature inspired the development of the reference-back task. We agree that it may still include some confounds but the reference-back provides the advantage of allowing dissociable measurements between processes (e.g., input gating, updating, maintenance and matching) that the n-back task does not dissociate. This is because the reference-back task includes sequences of maintenance trials that serve as baseline to the updating trials in terms of frequent perceptual changes and the matching decision required on every trial. Importantly, input gating is triggered by switching between updating and maintenance trials which can also be thought of as switching between a 1-back and a 0-back. The new version of the task, the reference-back-2, is a hybrid between the SIR2 (motivated by the model) and the n-back, and is therefore better tailored for probing selective gating operations. We do not down that an RNN can perform the n-back when trained to do so, but we think it is unlikely that without trainable gating policies as in PBWM (or LSTM etc), the RNN would likely not be able to transfer this knowledge to other types of stimuli and context, or to easily switch between different n-back levels.

- Relatedly, in the Discussion (page 22, Line 485), the authors claim that the reference-back-2 task can assess prioritization without being contaminated by removal. However, when a certain category is prioritized (say, letter) and the item needs to be updated (X -> O), the old item still needs to be removed.

We thank the reviewer for this point. The common probing of prioritization with retro cuing (when it is reliable) elicits prioritization of the cued item and removal of the un-cued item(s). An important distinction of the reference-back-2 task is that the currently irrelevant item (un-selected by the gate) still requires active maintenance for future trials so selection of a representation is decoupled from the removal of the *other* representation. Indeed, prioritization of category and action were stronger in updating trials but the effects were observed in maintenance trials as well. Importantly, the evidence for prioritization (see updated Fig. 5) started to emerge at an early time window that is highly likely to precede any updating or removal (e.g., before 300ms). Presumably, the updating and removal of the stimulus did not precede the output selection, otherwise no matching decision between probe and WM would be possible (although it is still likely that these processes occurred somewhat in parallel).

- Figure colors: The authors tend to use very similar colors in figures which are hard to distinguish. For example, Figure 1 uses light and dark orange, and bar graphs in later figures were colored with slightly different shades of green, blue or orange. Maybe consider using colors with higher contrast.

- Figures 5 and 8 have a lot of bar graphs that are difficult to grok.
a. All the figures look kind of similar and it is not easy to tell what metrics and what kind of comparisons they are showing without perusing the figure captions and main text. The authors can consider using letter indices (A, B ..) instead of “left corner”, “top” to refer to specific graphs. Consider using titles and sub-titles for columns/rows to refer to groups of figures, e.g., for the 4 RT graphs and the 4 Error Rate graphs.
b. Even though in each group there are 4 figures with 4 bars each, upon a closer look, there are only 8 distinct bars (repeat, input, output, resp, input+output, input+resp, output+resp, and all-sw), with a lot of redundancy. I understand the authors want to showcase the separate interactions, but maybe they can condense the plots into 1 or 2, or think of a clearer and more concise way to present the data.
c. Consider using asterisks and brackets to indicate significant main effects and interactions: right now it’s hard to see what the authors are trying to show us. Also it is not easy to locate the “under-additive interactions” just by looking at the bars. Highlight them in some way.
All the figures in the ms were modified to increase interpretability.

- Figure 5 (page 16):
a. Panel a is not clear. It seems to be a cartoon/schematic as we don’t know what the GLM mask is for (category or response). Moreover, the text around the heatmap is too small to decipher, the figure titles are not informative, and the axes are not labeled. And, where is the “red rectangle” that the caption referred to Line 369)?
b. In Panel b, it might be better if authors can have a significance bar that indicate time frames with significant differences between switch and repeat conditions. And why the big swaths of white on the bottom row?
All the figures in the ms were modified to increase interpretability. The temporal dynamics are based on the time points where the mask captured by the GLM was significant so that is why the plot is not always continuous. We made this point clearer in the figure and in the figure caption.

- Both Figures 6 and 7 show that the HDDM fits the data well. Maybe one figure is sufficient and move the other one to the Supplemental Materials?
- Page 20: Line 440 says “drift rate was also allowed to vary by condition” whereas Line 449 says that the decision threshold holds “while accounting for impact of difficulty on drift rate.” I thought “accounting for …” means to “hold constant”? Please clarify

The aim of the DDM analysis was to test the important extension of the PFC-BG model that not only response conflict is manifested by increase in threshold but also cognitive conflict recruits the same control mechanism of raising the threshold (see detailed answered in the next comment).

To empirically test the importance of the threshold parameter in describing the data we also allowed the drift rate to vary by condition. We explain this point at the end of the discussion:

“Notably, these threshold effects were obtained even though drift rate was also allowed to vary by condition (see supplementary Fig. S3). In the DDM, (lower) drift rates can also capture slowed RT when a condition is more difficult, as opposed to a control mechanism. Accordingly, we did observe that drift rates were slower in conditions that required more cognitive processing to succeed: updating vs. maintenance trials and switches at the cognitive level vs. switches at the response level. In both cases additional cognitive processing is required: updating trials require replacing WM content in addition to accessing previous content for responding, and switches at the cognitive levels are known to be more difficult than simple response switches (e.g., [54]). These findings boost the interpretation that gate switches at any given level are specifically related to elevated decision thresholds, while accounting for impact of difficulty on drift rate.”

Why is drift rate negative? Does this correspond to a reduction of drift rate? Because drift rate also increases as it goes from high to low (response) level, why is the story mainly about the decision threshold but not about drift rate?

The reported posterior mean of the parameters is relative to the base line condition in our task, which was also the easiest condition: maintenance with repeat in all gates. Thus the negative parameter only means that these parameters are less positive than the easy condition, not absolute negative drift. To make the results section more concise and interpretable we have now plotted drift rates as positive and added an explanation for this choice of presentation in the figure caption. We also moved the drift rate results to the supplementary material, given our focus here and our hypotheses.

Regarding the focus on the threshold and not the drift rate, we elaborated on the specific prediction that can be tested specifically with modeling the RT distributions (see page 15) and elaborated on the unique effects that were observable by this model. In particular the PBWM model predicts that conflict at higher levels leads to an increase in motor response threshold, given that they recruit the same control mechanism. In contrast, the drift rates are predicted to vary according to any model in which some task conditions are easier than others. We elaborated this with an example in the discussion.

“Gate switching at any level increases decision threshold.

The behavioral results described above revealed that switches at any level of gating were related to increased RT cost. To further decompose separable cognitive processes that give rise to such changes in RT, we leveraged the drift diffusion model (DDM). As noted in the introduction, various studies have suggested that dorsomedial frontal cortex detects response conflict (which is elevated following switches) and it recruits the mPFC-STN network that increase the effective decision threshold [46-49]. In the DDM, changes in the threshold are used as a cognitive control mechanism that regulates the speed-accuracy tradeoff, with larger thresholds related to slower but more accurate decisions. Importantly this threshold parameter is separable from impacts of other parameters (such as the rate of evidence accumulation or “drift rate”, whereby a slower drift rate would lead to slower but less accurate decisions). Thus to properly test out PBWM prediction, we relied on the joint distribution of RTs and error rates and fit the DDM.

To illustrate the functional importance of the decision threshold on optimal behavior during conflict, consider the following example. Imagine you are driving in the morning from home on your routine way to work but this morning you have to first stop by the dentist for a scheduled appointment. You get to the intersection near your work place where you always take a left to get to work so the visual information of the familiar intersection gives rise to a prepotent response to turn left. However, now you also need to consider your goal in WM that you first need get to your dentist appointment. You then need to retrieve the directions to the dentist from memory before you can decide if it requires going left or right. This situation is referred to as a response conflict because the incorrect habitual response to turn left competes with the context-dependent correct response, which may be to turn right. Such conflict is thought to be managed by an interaction between the medial PFC and the STN that can temporarily pause the response gate by sending diffused excitatory projections to the GPi through a “hyperdirect” pathway that bypasses the striatum. This excitatory projection to the GPi, temporarily suppresses the response gate [66-67] preventing it from selecting any response including the premature response that was activated based on the visual input alone. Neural models and previous empirical findings demonstrated that such response conflict is reflected by a mid-frontal EEG signature that triggers the increase of the decision threshold on the response gate via this mPFC-STN network [33, 56, 68-72]. The additional time provided by the increased decision threshold, allows a more cautious and deliberate response selection that also considers the evidence provided by WM information. An important extension of previous findings that we aimed to test here was whether information selection to or in WM by the input and the output gate, also manifests as a cognitive conflict that delays responses until the WM conflict is resolved, as predicted by hierarchical models of PFC-BG [12].”

The description of the results was also modified (page 17):

“…switches at each independent level of gating (input, output, and response) were related to increased decision thresholds (see Fig 7 for statistics). Notably, in updating trials, such effects were incrementally larger when switches were closer to the response level. This result supports the notion that response conflict has preferential impact on motor decision threshold, but that switches in higher level gates can nevertheless recruit the same process.”

In the discussion we mentioned that:

“We found that switches at any gating level were related to increases in the estimated decision threshold. This finding builds on previous studies showing that switches or response conflict gives rise to increased threshold via PFC-BG mechanisms [46, 48,49], but extends it to confirm the hierarchical PFC-BG model prediction that such effects hold for switches at more cognitive levels [12]”.

- Linking gating and prioritization: seems like looking at the similarity of EEG pattern to the masks that care about the representation (category or response) is a weird way of decoding… The word “decoding” gets used a lot, and often not very precisely. Here, you are not decoding an item’s identity per se, but rather looking at how much the brain signal looks like the pattern that is distinguishing the items?

We thank the reviewer for this point and we agree. We replaced throughout the ms the term ‘decoding’ with ‘neural index’ where increase in the neural index means that the discriminability between the two candidate representation for selection by the gate increases. Such increase in discriminability is a proxy of prioritization where one representation is presumably more active than the other.

- P. 23, line 509: should it be “than” instead of “then”?

Fixed.

--------------------------------------------------------------------------------------------------------------------

**Reviewer #2:** PCOMPBIOL-D-21-00030

Analogous computations in working memory input, output and motor gating:
Electrophysiological and computational modeling evidence

By Rac-Lubashevsky and Frank

SUMMARY
This study utilized behavior paradigms, EEG, decoding analyses of EEG signals, and a DDM to evaluate the PBWM (prefrontal cortex basal ganglia working memory) model that posits working memory involves a hierarchy of selective input, output, and response gating operations. They adapted the reference-back task, which had previously been used to study PFC-BG connections related to input gating, to require subjects to track two independent categories over trials, which allowed the experimenters to require input gating while orthogonally manipulating output gating. Univariate analysis of EEG data showed a confirmation of their hypothesis that neural correlates of different gating types evolve dynamically and have similar behavioral effects. They found that motor responses were enhanced following responses switches, and that the decodability of the relevant WM category was enhanced after an ‘output gate switch’. This could result from a change in the “active neural trace” or an increase in the accessibility for “read-out”. Regardless, they argue that the cognitive gating operations are implemented by similar cortico-striatal circuits that have been linked to motor gating operations. The authors then used computational modeling, HDDM (hierarchical Bayesian estimation of DDM parameters), to corroborate their findings. They found that switches at any gating level related to increases in the decision threshold. Finally, the researchers also described the “under-additive” interactions when more than one type of switching was needed. The results suggest that actions selection is at least partially parallel, and that decisions are processed even while the identity of the cognitive rule is uncertain.

EVALUATION
This study is well motivated and addresses and important question about the neural mechanisms of working memory. The reference-back task paradigm is an appropriate choice and well suited for the EEG approach taken. The addition of the DDM modeling allowed them to support and extend their findings. My biggest area of concern is in how the data are presented and how the results are explained (e.g., in Figs 3, 6, 7, and 8). The plots are too cluttered, with insufficiently contrasting colors for different conditions, and without any visual markers of statistical significance for key findings. Also, while the **DDM modeling was used to support their findings, its inclusion is not sufficiently justified and should be more clearly motivated.**

Thank you for the comments and evaluation. We improved the presentation of the modeling and the data, with all new figures, as elaborated below.

MAJOR CONCERNS

1. THEORY EXPLANATION. The description of the PBWM architecture in Figure 1 is quite confusing. There are so many different uses of colored arrows, colored arrow heads, colored letters, yellow squares, etc. It is really challenging to follow and does not serve the authors well in explaining the proposed gating model. This figure should be reconceptualized and more clearly explained to the reader.

The schematics of the PBWM architecture were modified substantially for clarity. Color coding was also added as suggested in a later comment, and the figure caption was edited to facilitate understanding of the model.

**“Figure 1. Schematic of the PBWM architecture for canonical gating operations**.

The gating system is composed of three core circuits that are hierarchically arranged from left to right. For illustration the operations needed to solve the task used in this paper are shown. At the beginning of a trial, the memory layer actively maintains the information from the previous trial (e.g., “O” and “%”). Sensory information (e.g., the “X” in the visual input) is transiently represented in a prefrontal cortex layer (labeled “PFC-in”). The input gate controls whether the new visual information (e.g., “X”) is updated to be maintained in a PFC memory layer, and if so, to which address or anatomical “stripe” within that layer (selective gating). The colored frame in the visual input represents a task cue signifying the updating policy that should be taken by the input gate. In this example, the red frame indicates that memory should be updated, so the input gate selectively updates the right PFC stripe (corresponding with the letter category, replacing the “O” in memory with “X”). The output gate controls which memory representations is prioritized in PFC-out (e.g., a deep layer of the PFC) to influence subsequent processing (e.g., here the relevant representation in memory is “O” and not “%”, given that the input to be compared is in the letter category). The response gate controls which motor response to select (e.g., here “same” (S) or “different” (D)) in posterior prefrontal layer (labeled here PMC), by comparing the output-gated memory representation (“O”) with the current visual input (“X”). Gate switching at the output and response gates are reflected by the change in the active stripe between the previous trial (t-1) and in the current trial. **(b)** An example of a maintenance trial. The blue task cue indicates that the appropriate updating policy is maintenance, and thus sensory information (e.g., “X”) is prevented from being updated in PFC memory (the gating signal from the BG is therefore a No-Go, indicated by the red BG). Gate switching in the input gate corresponds with moving between updating state (as shown in a) and maintenance state (as shown in b). Yellow squares indicate active stripe selected by the corresponding gate. The orange square represents the newly updated stripe in memory. The predicted temporal order of the gating loops is depicted at the bottom right of the figure with input and output gating starting roughly at the same time continuing in parallel (indicated in orange) with output gating terminating later (serial termination indicated in grey). The response gate is expected to initiate with some delay due to conflict at upper levels, and to terminate even later than the output gate.”

2. DATA PRESENTATION. The presentation of the data severely limits their interpretability and impact. For example, Figs 3, 6, 7, and 8 present large piles of data that are insufficiently labeled, poorly differentiated by subtle color variations (dark green vs. light green), and poorly organized so that interpreting the results is challenging if not nearly impossible. I cannot emphasize how much the use of “dark green / light green” contrasting colors makes these plots even worse. This comes up often and cannot be overlooked. Many subplots in these figures lack titles or other organizational signposts to help explain the results. The Results section is littered with statistical comparisons and significance results which are not found in the figures referenced. This puts the onus on the reader to “figure things out” which is not acceptable. Also, bar plots are presented without the underling individual data points, and often it is not clear if the error bars shown are STD, SEM, or 95% CI.

We thank the reviewer for this point and we agree. All the figures in the ms were modified to increase interpretability. The figure captions were edited as well to describe all the key aspects of the figures.

3. MODEL JUSTIFICATION. The use of the computational model is not sufficiently justified in the paper. It is unclear what value is added by the modeling that was not already found from the EEG results. I believe their *is* value added, but this needs to be spelled out in detail for the reader. In the Introduction, the authors say that the DDM can approximate BG dynamics, which is helpful, but not sufficient motivation for the added complexity (and inherent assumptions) of the model. The indecipherable Figure 8 compounds this issue by obscuring the results of the model. The metrics in this figure are explained in a single line: “We thus assessed whether gate switches at all levels of the WM hierarchy are quantitatively related to adjustments in decision threshold (thought to reflect cognitive control, thus related to slower but more accurate responding), or whether any such effects could be attributed to alterations in drift rate (thought to reflect increased difficulty and thus slower and less accurate) (Fig. 8).” This needs to be fleshed out more, especially for readers who are less familiar with DDMs and how their parameters may correspond to different neural mechanisms. Finally, near the end of the Discussion, the authors note a limitation of the HDDM framework that opens up more questions: “other models should be considered in future work (including those with time-varying drift rates and/or boundaries)”. It is unclear why their current model cannot address these issues.

We thank the reviewer for pointing out that the motivation for using the HDDM was not clear. We elaborated on the specific prediction that can be tested specifically with modeling the RT distributions using the DDM (see page 15 in clean ms) and we elaborated on the unique effects that were observable by this model including the important extension of the PFC-BG model that not only response conflict is manifested by increase in threshold but also cognitive conflict recruits the same control mechanism to raise the threshold:

“Gate switching at any level increases decision threshold.

The behavioral results described above revealed that switches at any level of gating were related to increased RT cost. To further decompose separable cognitive processes that give rise to such changes in RT, we leveraged the drift diffusion model (DDM). As noted in the introduction, various studies have suggested that dorsomedial frontal cortex detects response conflict (which is elevated following switches) and it recruits the mPFC-STN network that increase the effective decision threshold [46-49]. In the DDM, changes in the threshold are used as a cognitive control mechanism that regulates the speed-accuracy tradeoff, with larger thresholds related to slower but more accurate decisions. Importantly this threshold parameter is separable from impacts of other parameters (such as the rate of evidence accumulation or “drift rate”, whereby a slower drift rate would lead to slower but less accurate decisions). Thus to properly test out PBWM prediction, we relied on the joint distribution of RTs and error rates and fit the DDM.

To illustrate the functional importance of the decision threshold on optimal behavior during conflict, consider the following example. Imagine you are driving in the morning from home on your routine way to work but this morning you have to first stop by the dentist for a scheduled appointment. You get to the intersection near your work place where you always take a left to get to work so the visual information of the familiar intersection gives rise to a prepotent response to turn left. However, now you also need to consider your goal in WM that you first need get to your dentist appointment. You then need to retrieve the directions to the dentist from memory before you can decide if it requires going left or right. This situation is referred to as a response conflict because the incorrect habitual response to turn left competes with the context-dependent correct response, which may be to turn right. Such conflict is thought to be managed by an interaction between the medial PFC and the STN that can temporarily pause the response gate by sending diffused excitatory projections to the GPi through a “hyperdirect” pathway that bypasses the striatum. This excitatory projection to the GPi, temporarily suppresses the response gate [66-67] preventing it from selecting any response including the premature response that was activated based on the visual input alone. Neural models and previous empirical findings demonstrated that such response conflict is reflected by a mid-frontal EEG signature that triggers the increase of the decision threshold on the response gate via this mPFC-STN network [33, 56, 68-72]. The additional time provided by the increased decision threshold, allows a more cautious and deliberate response selection that also considers the evidence provided by WM information. An important extension of previous findings that we aimed to test here was whether information selection to or in WM by the input and the output gate, also manifests as a cognitive conflict that delays responses until the WM conflict is resolved, as predicted by hierarchical models of PFC-BG [12]..”

The description of the results was also modified (page 17 in clean ms):

“…switches at each independent level of gating (input, output, and response) were related to increased decision thresholds (see Fig 7 for statistics). Notably, in updating trials, such effects were incrementally larger when switches were closer to the response level. This result supports the notion that response conflict has preferential impact on motor decision threshold, but that switches in higher level gates can nevertheless recruit the same process.

Relatedly, parameter estimates revealed under-additive interactions (Fig 7) that mirrored those described in behavioral summary statistics above, and are also consistent with the EEG findings that gate switches overlapped in time. Specifically switching both input and output gates increased threshold to a similar level as switching only one gate, again suggesting that input and output gating in WM are two independent selection mechanisms that can be managed simultaneously. Furthermore, in updating trials, the threshold was most strongly elevated by response gate, such that any additional conflict at the cognitive level (by input or output switch) did not further elevate the threshold much when response conflict was present. Conversely, in maintenance trials, input gate switching (i.e., switching to a maintenance policy by closing the gate) had the strongest impact on threshold whenever another gate was switching with it. We speculate about potential mechanisms for this effect in the discussion.”

In the discussion we mentioned that:

“We found that switches at any gating level were related to increases in the estimated decision threshold. This finding builds on previous studies showing that switches or response conflict gives rise to increased threshold via PFC-BG mechanisms [46, 48,49], but extends it to confirm the hierarchical PFC-BG model prediction that such effects hold for switches at more cognitive levels [12].

Furthermore, by comparing the impacts of threshold adjustment when multiple gates are switching, the HDDM analysis provided some further insight into the observed parallel processing patterns in RT and in the EEG. First, threshold adjustments were under-additive, with largest adjustments dictated predominantly by switches in response gate for updating trials, but by switches in input gating for maintenance trials (i.e., the need to close the gate to prevent updating). We speculate that closing the gate over WM might also recruit the same prefrontal sub-thalamic pathway thought to be involved in transient response inhibition (e.g., [68, 81]), therefore switching to a maintenance mode might have a larger impact on motor gating than switching to an updating mode. A limited finding that is consistent with this hypothesis is the previous observation in the reference-back task [27] that input switching to maintenance (gate closing) was marked by a mid-frontal EEG signature that has previously been associated with the triggering of the mPFC-STN network during response conflict [56; 68]. Future research should test the possible similarities in the neural mechanisms that are triggered by response inhibition vs cognitive inhibition in WM.”

Regarding the limitation of the HDDM model used in the ms, to our knowledge this is the first attempt to use quantitative computational modeling to understand the decision dynamics of gated WM tasks such as the reference-back task that involves high level cognitive decisions such as input and output gating. As such we felt that for clarity of interpretation it would be most useful for cognitive scientists to evaluate the results in the context of the well-established DDM, for which the threshold is commonly interpreted and distinguished from other parameters, and contextualized by the various findings linking it to the mechanism of interest in PFC and BG. Of course, it is essential that to interpret the parameters that the model provides a reasonable fit to the RT distributions and error rates, and we did verify in the ms that it did. Our comment on the limitations thus simply amounts to noting that further studies might identify other decision dynamics that account for more nuanced features of the data, but it is unclear whether those other more complex (and computationally expensive to fit) models would be justified for this task.

4. WHY THE BG? The only evidence presented that supports the supposition that the BG is involved in the gating mechanisms being targeted here is based on the HDDM model, which itself is built on assumptions. There is no direct evidence in the EEG data of the BG involvement, of course, because of the ‘inverse problem’ and the impossibility of localizing the source of EEG signals. However, there are analytic approximations (e.g., Loreta variants) for localizing electrical potentials from scalp recordings. Some approach like this should be used attempted to justify the neural circuit claims of the PBWM model. Relying on assumptions of the HDDM model alone to describe the involvement of the BG is insufficient without a significant amount of further explanation and justification.

We thank the reviewer for allowing us to clarify this important point. The involvement of BG in WM and response gating is well documented in previous studies that are cited in the ms (e.g., 6-12,14,18,30,48). The purpose of the current work was to test the decision dynamics of the PBWM gating model which were not tested in previous work. We are not making any claims that the current data index BG activities, and we don’t feel that would be convincing with source localization either. The reason we mention the BG is because of its posited role in gating in the model, and the specific predictions about how the BG mechanism leads to similar response cost following switches at all levels. We focused on providing new insight on the temporal order and computational principles of the gating decisions. The new design used in the ms, the reference-back-2 task provided the means to empirically test the most central prediction of the model, that cognitive and motor gating share analogous computational principles.

MINOR CONCERNS

a. Fig. 5 notes that it contains a “red rectangle” to indicate the sliding window for the similarity index, which is not present. That section of the figure, panel a, is confusing, with impossibly small labels, and overall hard to interpret.

b. The concept of “under-additive” interaction should be better explained in the text. This is a concept that may not be self-evident and as a reader I had to spend time try to understand the specific point made in the context of this paper.

We thank the reviewer for pointing out that more clarification was required. We edited to prediction section in the introduction and added a paragraph to discuss the prediction of parallel processing in the gating system. In the new paragraph we also added an explanation for the meaning of under-additive interaction:

“The evidence for parallel processing across levels of information is also well documented in task switching literature when task sets and responses needed to switch [64-65] but also when context and items in WM had to both switch [66]. Parallel processing in these cases were captured by under-additive interactions reflecting that the cost of switching two levels of decision was less than the sum of their individual durations.”.

Also, Figure 3 and 7 were modified to ease on the interpretability of these interactions.

c. Figs. 6 and 7 could easily be combined into one smaller plot. The display of every level in multiple subplots is unnecessary when the main take away is a lack of difference.

d. Fig. 5b is using SEM, not 95% Cis, for error shades, so significance between conditions needs to explicitly marked in the figure, else the errors should be switched to 95% CIs.

e. Fig. 1 could be improved by following conventions from other model diagrams, such as an LSTM unit: <https://www.researchgate.net/figure/The-structure-of-the-Long-Short-Term-Memory-LSTM-neural-network-Reproduced-from-Yan_fig8_334268507>.

We thank the reviewer for these points and we agree. All the figures in the ms were modified to increase interpretability. The figure captions were edited as well to describe all the key aspects of the figures.
